# Polymeric Microneedles Enhance Transdermal Delivery of Therapeutics

**DOI:** 10.3390/pharmaceutics16070845

**Published:** 2024-06-22

**Authors:** Hiep X. Nguyen, Thomas Kipping, Ajay K. Banga

**Affiliations:** 1 Department of Pharmaceutical Sciences, College of Pharmacy, Mercer University, Atlanta, GA 30341, USA; 2 Faculty of Pharmacy, Phenikaa University, Yen Nghia, Ha Dong, Hanoi 12116, Vietnam; hiep.nguyenxuan@phenikaa-uni.edu.vn; 3 MilliporeSigma, a Business of Merck KGaA, Frankfurter Strasse 250, 64293 Darmstadt, Germany

**Keywords:** fabrication, characterization, polymeric microneedles, microchannels, methotrexate, transdermal delivery

## Abstract

This research presents the efficacy of polymeric microneedles in improving the transdermal permeation of methotrexate across human skin. These microneedles were fabricated from PLGA Expansorb^®^ 50-2A and 50-8A and subjected to comprehensive characterization via scanning electron microscopy, Fourier-transform infrared spectroscopy, and mechanical analysis. We developed and assessed a methotrexate hydrogel for physicochemical and rheological properties. Dye binding, histological examinations, and assessments of skin integrity demonstrated the effective microporation of the skin by PLGA microneedles. We measured the dimensions of microchannels in the skin using scanning electron microscopy, pore uniformity analysis, and confocal microscopy. The skin permeation and disposition of methotrexate were researched in vitro. PLGA 50-8A microneedles appeared significantly longer, sharper, and more mechanically uniform than PLGA 50-2A needles. PLGA 50-8A needles generated substantially more microchannels, as well as deeper, larger, and more uniform channels in the skin than PLGA 50-2A needles. Microneedle insertion substantially reduced skin electrical resistance, accompanied by an elevation in transepidermal water loss values. PLGA 50-8A microneedle treatment provided a significantly higher cumulative delivery, flux, diffusion coefficient, permeability coefficient, and predicted steady-state plasma concentration; however, there was a shorter lag time than for PLGA 50-2A needles, base-treated, and untreated groups (*p* < 0.05). Conclusively, skin microporation using polymeric microneedles significantly improved the transdermal delivery of methotrexate.

## 1. Introduction

Compared to conventional administration routes, transdermal delivery offers distinct advantages: (i) it circumvents gastrointestinal-induced drug degradation, (ii) bypasses the first-pass hepatic metabolism for enhanced bioavailability [1,2], (iii) provides sustained and controlled drug delivery, (iv) enables non-invasive and painless drug administration without risk of bleeding or infection [2], and (v) enhances the patient convenience, compliance, and acceptability [1]. Despite these benefits, transdermal drug delivery is restricted to a few compounds, which typically possess high potency and moderate lipophilicity, such as small molecules (<400 Da), and they melt at relatively low temperatures. Some examples include scopolamine for motion sickness, nicotine for smoking cessation, nitroglycerin for chest pain, fentanyl for severe pain, estradiol and testosterone for hormone therapy, lidocaine for local anesthesia, clonidine for high blood pressure, oxybutynin for overactive bladder, and diclofenac for pain and inflammation [3,4,5,6]. Delivering therapeutic agents through the skin (transdermal delivery) faces a significant hurdle: the skin’s natural barrier function. The uppermost and lipophilic layer of the skin, the stratum corneum, serves as the primary barrier to block the passive diffusion of most therapeutics [7]. Researchers have continually explored a repertoire of novel physical techniques to circumvent limitations in drug penetration, such as microneedles [8,9,10], iontophoresis, ablative laser [11], sonophoresis [12], and microdermabrasion.

Methotrexate (MTX) is an antagonist of folic acid, inhibiting the dihydrofolate reductase enzyme and DNA synthesis [13]. Due to profound anti-neoplastic and antirheumatic properties, MTX is a potent treatment for specific neoplastic conditions at high doses. Conversely, at lower doses, MTX effectively manages severe psoriasis and rheumatoid arthritis [14,15,16]. MTX exists in the pharmaceutical market as tablets (oral route) and intramuscular/intravenous injections (parenteral route) [16]. Nevertheless, the systemic administration of MTX is associated with a substantial risk of side effects, including hepatotoxicity (the principal toxicity and serious long-term adverse effect), myelosuppression, stomatitis, nausea, and vomiting [15,17,18]. The oral administration of MTX is hampered by limited gastrointestinal absorption, partial metabolism by intestinal bacteria, and inability to reach the psoriatic lesions [19]. Thus, the drug bioavailability is limited to approximately 40% [1]. The parenteral delivery (subcutaneous injection) of MTX receives poor acceptability, inconvenience, and patient incompatibility, as this route is associated with needle phobia and trained healthcare givers [20]. Subcutaneous tissues, abundant with loosely packed fat cells and well-vascularized, cause MTX to be cleared rapidly [21]. Therefore, subcutaneous injection resulted in insufficient delivery and an accumulation of MTX in the psoriatic lesions [22]. Hence, an alternative route of drug delivery is necessitated to enhance the therapeutic efficacy and mitigate the adverse effects of MTX.

Topical and transdermal delivery might be the preferable route to administer MTX to minimize the drug’s first-pass hepatic metabolism and reduce the severe adverse effects of systemic delivery [23]. MTX could be used as a topical treatment for localized rheumatic, dermatologic, and skin diseases [24,25]. At the same time, a clinically therapeutic dose of MTX delivered transdermally presented an efficacious therapeutic avenue for acute lymphocytic leukemia [2]. However, the physicochemical properties of the drug inherently restrict its capacity to permeate the stratum corneum. These limitations stem from its hydrophilicity (Log *p* = −1.85), negative charge at physiological pH, and moderately large molecular weight [1,2,26]. The inadequate permeation of MTX is the primary reason for the lack of clinical studies of topically and transdermally delivered MTX [27]. Several groups have managed to improve the percutaneous absorption of MTX through the skin by developing MTX-loaded formulations such as microemulsions [28] and liposomes [29] and employing physical enhancement technologies [11,28,30], such as iontophoresis [12,28,31], electroporation [25], microneedles [11,16], and ablative laser [11,32].

Recently, microneedles have emerged as a highly compelling platform for the transdermal and topical delivery of diverse therapeutics (including small molecules, macromolecules, and nano/micro-particles [33]), garnering significant interest due to their potential for safe and efficacious drug delivery. Microneedles, designed for minimally invasive drug delivery, are miniature, needle-shaped devices that range in length from 250 μm to 1500 μm [9,10,11]. After insertion into skin tissue, microneedles transiently disrupt the stratum corneum, generating a network of hydrophilic microchannels within the skin. These micron-sized conduits, filled with interstitial fluid, facilitate enhanced drug permeation without causing bleeding, irritation, or pain sensation [34,35,36]. Microneedles are engineered to create microchannels within the stratum corneum and viable epidermis, strategically avoiding deeper penetration into the underlying dermis, where blood capillaries and nerve fibers are located [2,16,37]. Microneedle-created microchannels allow topically applied drugs to directly reach the dermis layers, significantly reducing the tortuous path length of drug permeation, thus effectively enhancing drug diffusion into the skin. The creation of hydrophilic microchannels by microneedle treatment has been reported to improve the transdermal permeation of MTX [2,16]. Interestingly, unlike conventional injection using hypodermic needles, microneedles could allow for self-administration [38].

Several groups have developed and fabricated microneedles for the transdermal delivery of MTX [39]. Recently, Du and coworkers fabricated dissolving microneedles containing MTX-encapsulated chitosan-coated mesoporous silica nanoparticles. The authors observed that microneedles could dissolve rapidly, allowing the drug-loaded nanoparticles to be precisely delivered to the skin lesion. MTX was then released continuously and gradually from the particles for at least a week to provide desirable long-term therapeutic effects in psoriasis treatment [40]. Research conducted by Dai and colleagues presented methacryloyl chitosan hydrogel microneedles that could release MTX sustainably and improve therapeutic efficacy [41]. Zhao et al. prepared tip-swellable dissolving microneedles to effectively carry and deliver MTX through the skin. The needles were reported to successfully penetrate porcine and psoriatic mouse skin, detaching and embedding the drug-loaded tips deeply in the skin, thus enabling more prolonged and sustained drug release (over 96 h) compared to subcutaneous injection and dissolving microneedles [19]. Rajendran et al. developed a biodegradable microneedle patch to deliver MTX sustainably and locally to arthritic joints of guinea pigs and found that MTX-loaded microneedles induced minimal immune response and provided sustained drug release for the rapid restoration of mobility and a significant decrease in inflammatory and rheumatoid markers at the affected joints compared to hypodermic injection [42]. Zhao and coworkers fabricated dissolving microneedles from hyaluronic acid and polyvinyl pyrrolidone K90 to deliver MTX transdermally. The authors revealed that microneedles markedly improved drug delivery and therapeutic performance compared to a cream formulation or intragastric administration. The pharmacological study indicated that MTX-loaded microneedles effectively mitigated paw swelling, reduced inflammatory response, alleviated synovium destruction, and delayed the progression of rheumatoid arthritis in rats [43].

A well-established FDA-approved material, poly(lactide-co-glycolide) (PLGA), offers a versatile platform for developing drug delivery devices and tissue engineering applications due to its lauded biocompatibility, biodegradability, and robust physical properties [44,45]. PLGA provides a controlled and favorable degradation mechanism and has been used in sustained drug delivery systems for various therapeutic agents [46,47]. PLGA hydrolyzes in the body to produce monomers of lactic acid and glycolic acid, which could be easily metabolized and eliminated via the Krebs cycle [48]. Consequently, the systemic toxicity of PLGA is negligible and insignificant. The degradation kinetics and mechanical properties of PLGA were demonstrably tailorable, enabling the design of precisely targeted delivery systems. PLGA has also been employed to fabricate solid and dissolving microneedles [11,49]. Specifically, Panda et al. used PLGA 50:50 and PLGA 65:35 with ratios of lactic and glycolic acids of 50:50 and 65:35, respectively, to fabricate FITC-dextran-loaded dissolving microneedles [50].

In the present study, we fabricated PLGA microneedles from PLGA Expansorb^®^ DLG 50-2A and 50-8A using the micromolding technique. Microneedle characterization encompassed dimensional analysis, chemical composition assessment, and mechanical uniformity evaluation. Precise measurements (microneedle length, base size, tip diameter, and inter-needle spacing) were obtained utilizing scanning electron microscopy (SEM). Fourier-transform infrared (FTIR) spectroscopy elucidated the chemical structure of both the PLGA granules and the fabricated microneedles. Finally, the mechanical uniformity of the microneedle array was rigorously assessed. A topical formulation of methotrexate (0.1% *w*/*w*) was prepared as a semisolid carbopol hydrogel. This formulation underwent comprehensive characterization to evaluate its physicochemical properties, including pH, density, drug content (assay), uniformity of drug distribution (content uniformity), and rheological behavior. The integrity and dimensions of microchannels generated by PLGA microneedles within human skin were evaluated using a battery of techniques, including dye binding, histological examination, scanning electron microscopy, pore uniformity analysis, and confocal laser scanning microscopy. Skin resistance and transepidermal water loss values of untreated and microneedle-treated skin were measured to examine the disruption of skin integrity and barrier function. Additionally, we conducted in vitro permeation and skin disposition experiments on the delivery of MTX into receptor solution and skin layers employing dermatomed human cadaver skin and vertical Franz diffusion cells. For the first time, we successfully fabricated solid polymeric microneedles from PLGA Expansorb^®^ DLG 50-2A and PLGA Expansorb^®^ DLG 50-8A. This demonstrated the possibility of using these grades of PLGA polymers for manufacturing biodegradable microneedles, which could effectively microporate skin tissue and significantly enhance transdermal drug delivery. Furthermore, the employment of human skin made the present research more relevant and applicable to future clinical studies on human subjects. In addition, we predicted optimal microneedle array dimensions of 1.08 sq·cm (PLGA 50-2A microneedles) and 0.34 sq·cm (PLGA 50-8A microneedles). These dimensions were strategically selected to achieve equivalent in vivo plasma levels of methotrexate achieved by oral administration.

## 2. Materials and Methods

### 2.1. Materials

Poly(lactic-co-glycolic acid) (PLGA) with a 1:1 lactide/glycolide ratio and terminal carboxylic acid groups (–COOH) was generously provided by Merck kGaA (Frankfurter Strasse, Darmstadt, Germany). Two PLGA grades were employed in this study: Expansorb^®^ DLG 50-2A with a molecular weight of 5–20 kDa and Expansorb^®^ DLG 50-8A with a molecular weight of 80–130 kDa. Methotrexate and Carbopol^®^ 980 NF were obtained from Sigma Aldrich, St. Louis, MO, USA, and Lubrizol Corporation, Wickliffe, OH, USA, respectively. Phosphate-buffered saline (PBS) was obtained from Fisher BioReagent (Fair Lawn, NJ, USA) at a concentration of 0.1 M and a pH of 7.4 ± 0.1. Eastman Kodak Company (Rochester, NY, USA) supplied the methylene blue dye. A 0.35% fluorescent solution (Fluoresoft^®^) was purchased from Holles Laboratories Inc. in Cohasset, MA, USA. The remainder of the reagents were of analytical grade. CuDerm (Dallas, TX, USA) supplied the D-Squame D101 stripping discs, while Dynarex produced the cotton-tipped applicators (Orangeburg, NY, USA). The Sylgard^®^ 186 silicone elastomer base and curing agent employed in this study were provided by Dow Corning Corporation (Midland, MI, USA). Micropoint Technologies Pte Ltd. supplied the master structure (a microneedle array consisting of 10 × 10 solid stainless-steel pyramidal-shaped needles with a 500 µm length, 150 µm × 150 µm base dimensions, and 500 µm inter-needle spacing) (Singapore). Human skin (posterior torso area of 48-year-old female) was acquired from New York Fire Fighter skin bank (Brooklyn, NY, USA). De-identified human skin was used under a protocol exempted by Mercer University Institutional Review Board (H0303041). Employing a calibrated material thickness gauge (Electromatic Equipment Co., Inc., Cedarhurst, NY, USA) with a measurement range of 0 to 25 mm, we determined the average thickness of 16 skin samples at 0.31 ± 0.06 mm. Until use, the skin pieces were cryopreserved in sealed containers in a freezer (–80 °C).

### 2.2. Hydrogel Preparation

Methotrexate (MTX) hydrogel was prepared using Carbopol 980 NF (Lubrizol Corporation, Wickliffe, OH, USA) as a gelling agent at a concentration of 1.5% *w*/*w*. Carbopol powder was slowly mixed at 300 rpm and dissolved in DI water overnight in ambient conditions and under continuous magnetic stirring at 300 rpm. Meanwhile, an aqueous solution of methotrexate was formulated at 5 mg/mL using propylene glycol as the solvent. This solution was subsequently incorporated into the carbopol slurry to achieve a final 1 mg/mL drug level, corresponding to 0.1% *w*/*w*. Notably, the formulation retained a concentration of propylene glycol at 20% *w*/*w*. The mixture was agitated at 300 rpm at ambient temperature for two hours. This solution was then neutralized to a pH of 5.5 ± 0.1 with triethanolamine to form a homogenous hydrogel.

### 2.3. Hydrogel Characterization

A pH/mV meter configured in mode 215 was used to determine the pH of MTX hydrogel (Denver Instrument, Bohemia, NY, USA). The pH meter’s clean and dry probe was immersed in a sufficient quantity of MTX gel. Real-time pH readings were captured and averaged, with the standard deviation reported for four replicates (n = 4). The hydrogel density was also determined to establish a weight–volume correlation. Precisely 10 µL of the gel, dispensed via a positive-displacement Pos-D™ pipette (Mettler-Toledo Rainin, Oakland, CA, USA), was weighed on an analytical balance to calculate the density (n = 10). In addition, the prepared gel underwent assay and content uniformity testing. Gel samples (10 µL) were removed from four distinct locations in the vessel and completely dissolved in 2 mL of phosphate-buffered saline at 10 mM and pH 7.4. After filtering through a 0.45 µm membrane, the sample was quantitatively analyzed utilizing an HPLC method.

A sand-blasted parallel-plate spindle (PP25/S-SN 33357, Anton Paar Germany GmbH, Ostfildern, Germany) was employed within a Rheoplus/32V.3.62 rheometer (Anton Paar) to characterize the rheological properties of the MTX hydrogel. The spindle configuration comprised a 2.4 cm diameter plate distanced from the station by a precisely controlled gap of 100 µm. Measurements were conducted at a constant temperature of 25 °C. A comprehensive oscillatory amplitude sweep test was then performed to delineate the product’s linear viscoelastic range (LVE). This test utilized a fixed angular frequency (ω) of 10 rad/s and systematically varied the applied strain (γ) from 0.001% to 100%. The dependence of shear stress, storage modulus (G′), and loss modulus (G″) on the amplitude of deformation was investigated and graphed. A precisely controlled oscillatory frequency sweep was performed at a fixed shear strain of 1%. The angular frequency range spanned from 0.1 to 100 rad/s. The complex viscosity (|η^*^|) and dynamic moduli (G′ and G″) were subsequently measured and plotted against the applied angular frequency. Moreover, a rotating rheometer was employed to generate a flow curve, elucidating the interplay between viscosity, shear stress, and shear rate across a range of 0–100 s^−1^. The viscosity was plotted versus the shear stress in the current investigation (0–200 Pa). Furthermore, the viscosity was determined with temperatures ranging from 5 to 65 °C with 20 °C increments. Thixotropic properties of the formulation were examined using a three-step oscillatory thixotropy analysis. Between two oscillation periods, a rotation phase was executed. For the first interval, the hydrogel was oscillated for 100 s at a fixed strain (1%) and angular frequency (10 s^−1^). During the second phase, the hydrogel was subjected to a vigorous shear rate at 3000 s^−1^ for one second. Subsequently, in the third period, the gel physical structure was allowed to recover for 300 s while maintaining a fixed strain of 1% and an angular frequency of 10 s^−1^. Our investigation involved the time-dependent acquisition of storage and loss modulus values, subsequently plotted across a 300 s duration.

### 2.4. Microneedle Fabrication

PLGA microneedles were prepared via a high-temperature, vacuum-based deposition process [49,51]. The selection of PLGA for microneedle fabrication was driven by its well-established biocompatibility profile, cost-effectiveness, and mechanical strength [52]. Furthermore, the risk for inadvertent skin retention of biodegradable PLGA microneedles could be alleviated by their safe disintegration and ultimate elimination from the body [7]. Microneedle mold fabrication commenced with the deposition of a pre-mixed polydimethylsiloxane (PDMS) solution (base to curing agent ratio of 10:1 *w*/*w*) onto a dry and clean master structure [7]. The coated structure underwent a two-step degassing process to eliminate entrapped air pockets. First, centrifugation at 4000 rpm and 25 °C for 30 min expelled air bubbles. Subsequently, a vacuum treatment (Binder GmbH, Tuttlingen, Germany) at 200 mbar and 25 °C for 30 min further ensured complete air removal. The micromold was then thermally cured at 100 °C for five hours to solidify the PDMS. After that, the cured PDMS mold was detached from the master structures and stored for future use to fabricate PLGA microneedles [7,51].

Pre-weighed quantities of PLGA Expansorb^®^ DLG 50-2A and DLG 50-8A polymers were directly placed onto the mold cavity. Subsequently, the mold was subjected to a high-vacuum treatment (200 mbar) within a dedicated vacuum oven (Binder GmbH, Tuttlingen, Germany) at 180 °C for 30 min. This thermal treatment ensured the complete melting of the PLGA granules. The molten polymer was drawn into the mold’s empty channels under continued vacuum, effectively eliminating any entrapped air bubbles within a 30 min timeframe. Following this process, the mold was taken from the oven and cooled down to ambient temperature for 10 min. The assembly was then briefly stored at −20 °C for 30 min to facilitate demolding. After that, the solidified PLGA microneedles were manually separated from the mold. The finished microneedle product was transferred to a desiccator and stored at 5 °C for subsequent experimentation. In total, twenty microneedle arrays of each group were fabricated and collected.

### 2.5. Skin Preparation

Cryopreserved human cadaver skin pieces, obtained from a sealed bag in a freezer at −80 °C, underwent a complete thawing process. The tissue was immersed in pre-warmed 10 mM phosphate-buffered saline at 37 °C for one min. After that, the skin tissues were dried using Kimwipes^®^ before being sectioned into uniform squares measuring 4 cm^2^. To simulate the cushioning effect of soft tissue underneath the skin, dermatomed human cadaver skin was secured in a planar orientation atop a quadruple stratum of Parafilm M^®^ (Neenah, WI, USA). Array base substrate without any microneedles (base-treated) and PLGA microneedles (microneedle-treated) were targeted and pressed onto the center of the skin sample for two min. A standardized application force, delivered either by thumb pressure or a spring-loaded applicator, ensured consistent microneedle insertion [53]. The microneedle-porated skin was then characterized prior to the commencement of the permeation experiments.

### 2.6. Ultrastructural Evaluation

Following microneedle application, the affected skin region was excised and mounted onto a Polysine^®^ microscope slide using a 1% *w*/*v* formalin solution for fixation. The tissue was then dried for 30 min in an oven at 50 °C and a vacuum level of 200 mbar. The dissected skin pieces were detached and affixed onto SEM pin stub mounts. The dust-free PLGA needles and the mounted skin tissues were then sputter-coated with gold to enhance conductivity, enabling detailed observation under a high-resolution Phenom^TM^ SEM system (Nanoscience Instruments Inc., Phoenix, AZ, USA) [8,53]. Leveraging SEM micrographs, we quantified the length, base size, tip dimensions, inter-needle spacing, and surface structure of PLGA microneedles. Furthermore, image analysis software (ImageJ, version 1.8.0) was employed to determine the surface area of the channels generated within the skin tissue (n = 10).

### 2.7. Chemical Characterization

Chemical characterization of PLGA polymer, pre- and post-microneedle fabrication, was performed using Fourier-transform infrared (FTIR) spectroscopy. The analysis was facilitated by a LabSolution IR software-equipped FTIR instrument (IRAffinity-1S, Shimadzu Corporation, Kyoto, Japan). Raw PLGA granules and finished microneedles were directly introduced into the FTIR chamber under ambient conditions. Subsequently, high-resolution spectra (4 cm^−1^) were acquired across a broad wavenumber range (400–4500 cm^−1^), encompassing 100 scans. This comprehensive spectral analysis enabled the interrogation of potential chemical modifications within the PLGA polymer, attributable to the microneedle production process.

### 2.8. Mechanical Analysis

Microneedle uniformity is paramount for consistent microchannel formation within the skin. The present investigation assessed the mechanical uniformity of fabricated microneedles. A model was constructed using four superimposed layers of parafilm (mean thickness: 0.14 ± 0.01 mm, n = 4). The microneedle patch was then employed to porate this layered construct. Following microneedle removal, the parafilm layers were collected and observed under a microscope (Leica DM 750, Leica Microsystems GmbH, Wetzlar, Germany). The size of twenty randomly selected pores on the uppermost layer was subsequently measured. Consistent pore dimensions served as a robust indicator of microneedles’ mechanical uniformity. Furthermore, the presence of all pores on the last layer corroborated the uniform needle lengths within the array.

### 2.9. Dye Binding Studies

To evaluate the effectiveness of PLGA microneedle-mediated skin microporation, the porated area was stained using a methylene blue solution (1% *w*/*v*). The dyed tissue was subsequently examined utilizing a mobile microscope (ProScopeHR Digital USB, Bodelin Technologies, OR, USA), as previously described [53,54]. Microscopic photographs of the skin tissue were captured to verify effective poration and determine the inter-pore spacing (n = 10) using image analysis software (e.g., ImageJ).

### 2.10. Histological Analysis

Histological evaluation of microneedle-generated microchannels was conducted using cryosectioning. Skin tissue porated by microneedles was first dyed with methylene blue solution. Subsequently, the tissue was embedded in Tissue-Tek^®^ optimal cutting temperature compound, frozen, and dissected at a thickness of 10 µm using a Microm HM505E cryostat (Southeast Scientific Inc., Dallas, GA, USA) [53]. Following hematoxylin and eosin staining, the sections were examined under a Leica DM 750 microscope for morphological assessment. Microscopic images were captured using a Leica DFC camera in tandem with the microscope.

### 2.11. Microchannel Uniformity

Calcine imaging with Fluoropore software was used to examine the consistency of microchannels (Altea Therapeutics, Atlanta, GA, USA). The microneedle-treated skin region underwent fluorescent staining with a 0.35% solution of Fluoresoft^®^ [53]. We collected and processed fluorescent photographs of the skin sample to determine the distribution of fluorescence intensity inside and around each pore. This fluorescence intensity was then used to calculate the Pore Permeability Index (PPI) value to indicate the consistency of calcein flux across individual pores.

### 2.12. Microchannel Depth

A confocal laser scanning microscope (Leica SP8 Microsystems, Deerfield, IL, USA) was employed to precisely quantify microchannel depth. The microneedle-porated skin region was covered with a 0.35% Fluoresoft^®^ solution. Excitation and emission wavelengths of 495 nm and 515 nm, respectively, were utilized on the computerized Leica SP8 system to evaluate channel depth and map the distribution pattern of calcein within the channels (n = 10). This approach leverages the high-resolution capabilities of confocal microscopy to provide a detailed characterization of the microchannels generated by the microneedles [53,54,55].

### 2.13. Skin Integrity Evaluation

Transepidermal water loss (TEWL) and skin electrical resistance measurements were employed to assess the level of skin integrity [12,53]. TEWL was assessed non-invasively (g/m^2^h, n = 4) using a Delfin VapoMeter (Delfin Technologies Ltd., Kuopio, Finland), which measured the water evaporation from skin and percentage relative humidity in the device chamber [2]. The skin electrical resistance was evaluated by transporting a current (a 100 mV alternating current at a frequency of 10 Hz) through a specific area of skin (n = 4). The skin resistance (R_S_, kΩ/sq·cm) was determined using Equation (1), analyzing the voltage reduction across the tissue (V_S_, mV) [11].

(1) $$R_{s}=\frac{V_{s}R_{L}}{A(V_{o}-V_{s})}$$

where

R_S_ denotes the electrical resistance of the skin (kΩ/sq·cm).
The voltage reduction across the tissue, as measured by the multimeter, is indicated by V_S_ (mV).
V_O_ represents the fixed circuit voltage at 100 mV.
R_L_ reflects the load resistance at 100 kΩ.
A indicates the permeation area of a Franz diffusion cells.

Compromised skin integrity was corroborated by a reduction in skin resistance and an elevation in the TEWL readings. Four microneedle arrays from each group were employed to treat four skin pieces separately in the skin integrity measurements (n = 4).

### 2.14. Permeation Studies

In vitro permeation experiments are often conducted to assess drug delivery into and through skin tissue [53]. The study technique was developed in light of our past research on methotrexate [11,12]. A Franz diffusion cell assembly (PermeGearV6 station, vertical orientation; PermeGear, Inc., Hellertown, PA, USA) was utilized for permeation studies (n = 4). The donor chamber, possessing an effective permeation area of 0.64 sq·cm and a 9 mm orifice, was loaded with 100 µL of methotrexate (MTX) hydrogel (0.1% *w*/*w*, corresponding to a total MTX quantity of 100 µg). The receiver chamber contained 5 mL of a 10 mM phosphate-buffered saline solution maintained at a physiological pH of 7.4. Throughout the experiment, the receptor solution was continually mixed at a speed of 600 rpm and kept at 37 °C. The skin tissue was positioned and oriented within the assembly, ensuring the stratum corneum was facing the donor compartment. The control group was identified as skin that had not been treated by microneedles. At 0, 1, 2, 4, 6, 8, 22, and 24 h, aliquots of receptor solution (300 µL) were removed and replenished with equivalent quantities of PBS. The collected samples were then subjected to quantitative analysis using the HPLC technique. The cumulative quantity of MTX permeating through the skin in vitro was calculated using Equation (2).

(2) $$Q=\frac{V}{A}C_{n}+\sum_{i=1}^{n-1}\frac{V_{i}}{A}C_{i}$$

where Q is the cumulative quantity of MTX (µg/sq·cm), C_n_ is the MTX concentration in the receptor medium at every sampling time (µg/mL), C_i_ represents the drug concentration of the sample (µg/mL). A denotes the effective diffusion area (sq·cm). V and V_i_ are the volumes of the receptor medium and the sample (mL), respectively [56]. The cumulative MTX permeation was then graphed against time to generate a permeation profile (n = 4). The lag time was defined as the x-intercept of the linear segment of the permeation graph (R^2^ > 0.95). The steady-state flux (J) of MTX permeation was determined using Equation (3) as the drug mass (m) traveling across a given cross-sectional skin area (A) over a specified time interval (t). The slope of this linear segment on the permeation profile represents the steady-state flux of MTX delivery.

(3) $$J=\frac{dm}{dtA}$$

The drug’s steady-state flux (J, µg/h) was employed to calculate the transdermal permeability coefficient (Kp, cm/h). This calculation incorporated the drug concentration within the donor compartment (C, µg/mL) and the skin’s surface area utilized for permeation (A), as displayed in Equation (4).

(4) $$K_{p}=\frac{J}{CA}$$

The diffusion coefficient (D, sq·cm/h) of MTX permeation was calculated from the lag time (t, h) and the skin thickness (h, cm) using Equation (5).

(5) $$D=\frac{h^{2}}{6t}$$

Employing Equation (6), we extrapolated the anticipated steady-state plasma concentration of methotrexate (C_ss_, µg/mL) based on the observed steady-state flux (J_ss_, µg/sq·cm/h), the effective permeation area (A, 0.64 sq·cm), and the inherent clearance of methotrexate from the human body (Cl).

(6) $$C_{ss}=\frac{AJ_{ss}}{Cl}$$

### 2.15. Skin Disposition Studies

Following the 2 h permeation studies, we quantified the distribution of methotrexate within distinct layers of the skin. A sequential approach was employed to remove residual MTX hydrogel from the donor chamber and skin surface. First, the skin surface was thoroughly swabbed with both dry (two) and receptor-wetted Q-tips (two). Subsequently, a standardized tape stripping protocol was implemented utilizing two stripping discs (D101). This ensured the efficient removal of any remaining drug on the skin surface. To isolate the epidermis for independent analysis, manual dissection with forceps separated this skin layer from the underlying dermis layer. Individually, these skin pieces were minced and collected into six-well plates. The skin tissue was extracted with a two-millimeter solution of methanol and PBS (1:1 *v*/*v*). This extraction process involved constant shaking at 100 rpm for 24 h at ambient temperature. Following extraction, the collected samples underwent filtration through a 0.2 µm membrane prior to quantification using an HPLC method [11,12]. The MTX content (µg/sq·cm) in the epidermal, dermal layers, and total skin was then quantified and graphed (n = 4).

The targeted skin delivery is depicted as topical selectivity (TS, %), which is calculated from the total quantity of drug delivery into the skin layers (skin disposition Q_s_, µg/sq·cm) and cumulative drug delivered into the receiver chamber at 24 h (Q_24_, µg/sq·cm), using Equation (7) [57].

(7) $$TS=\frac{Q_{S}}{Q_{S}+Q_{24}}\times 100$$

The total drug delivery (µg/sq·cm) is estimated as the sum of the drug quantity delivered into skin layers (Q_s_, µg/sq·cm) and the receptor medium (Q_24_, µg/sq·cm) [58]. Delivery efficiency (%) is defined as the fraction of the drug applied to the skin in the donor chamber that was efficiently delivered into and across the skin (total drug delivery).

### 2.16. Statistical Evaluation

Quantitative data were expressed as the average with standard deviation (SD) for all experimental groups (n ≥ 4). Statistical analyses were conducted utilizing Microsoft Excel software. Intergroup comparisons were performed using Student’s *t*-test and One-Way ANOVA with Tukey’s HSD post hoc test. A statistically significant difference was signified at a *p*-value threshold of less than 0.05.

## 3. Results and Discussion

### 3.1. Hydrogel Characterization

A comprehensive physicochemical evaluation of the methotrexate hydrogel was conducted. The hydrogel exhibited a pH of 5.50 ± 0.00 and a density of 1.00 ± 0.01 mg/μL (n = 4). Notably, microscopic examination at various magnifications revealed the complete dissolution of methotrexate within the hydrogel, with an absence of visible drug crystals. Quantitative analysis of the hydrogel formulation revealed a drug content of 0.11 ± 0.00% *w*/*w*. The low relative standard deviation of 2.14% across four distinct intra-container locations demonstrated content uniformity of methotrexate within the gel formulation.

**Figure 1:**
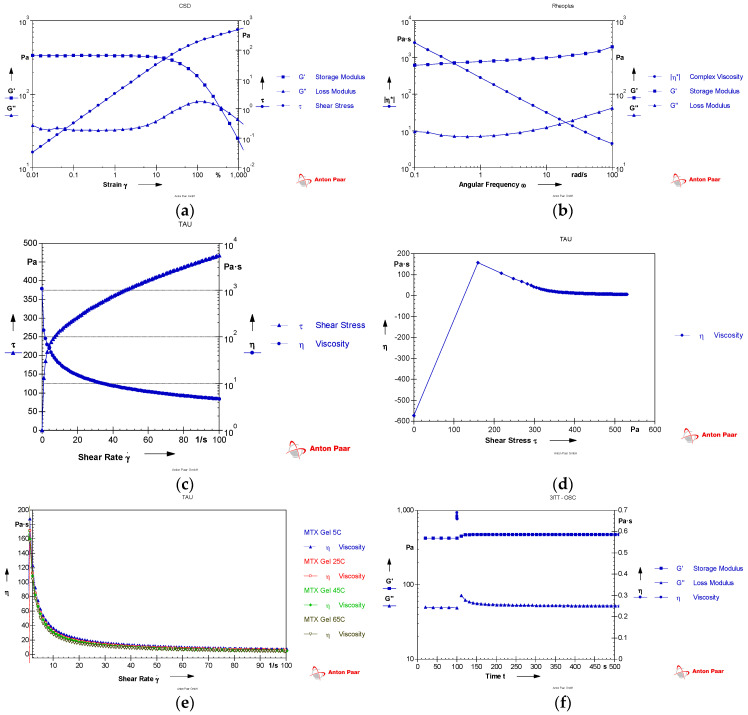
Characterization of methotrexate hydrogel rheology: (**a**) amplitude sweep, (**b**) frequency sweep, (**c**) flow curve, (**d**) yield stress, (**e**) temperature-dependent viscosity, (**f**) thixotropic behavior.

Regarding rheological properties, the storage modulus (G′) acts as a quantifier of a sample’s capacity to store elastic energy, effectively reflecting its rigidity or elasticity. Conversely, the loss modulus (G″) elucidates the energy dissipated within the sample, serving as an indicator of its viscous nature or fluidity. As illustrated in Figure 1a, G′ exceeded G″, indicating that the formulation structure was more elastic than viscous. The shear strain in the LVE range was measured to be between 0.01 and 1%. Consequently, strain (1%) was selected to perform the frequency sweep. As the applied shear strain exceeded the linear viscoelastic (LVE) region, both the storage modulus and loss modulus exhibited a decline, progressively signifying the disintegration of the gel network. The point of convergence between G′ and G″ (G′ = G″), also known as the flow point, marked the critical transition from a solid to a fluidic structure of the product. The gel structure was damaged with this shear strain. G′ and G″ increased in response to the increase in the angular frequency, while the gel’s complex viscosity declined (Figure 1b). Also, G′ consistently exceeded G″ across the investigated frequency range (0.1 to 100 rad/s). This observation underscored the intrinsic elastic nature of the formulated gel. Increasing the shear rate yielded a concomitant rise in shear stress while concurrently reducing the gel’s viscosity, as depicted in Figure 1c. This result indicated the gel formulation’s pseudoplastic nature and shear-thinning tendency, which helped to mitigate immediate injury when the product was rubbed on the skin [59]. Figure 1d shows that, as the applied shear stress progressively intensified, the viscosity exhibited a corresponding, yet initially rapid, increase to a peak value. Subsequently, the viscosity demonstrated a gradual reduction, ultimately reaching a plateau. When the viscosity of the gel was decreased, the gel’s thickening effect declined, and the physical state altered from semisolid to liquid. The point at which the gel structure was disturbed was characterized as flow initiation. This critical property influences the way to process, handle, and store the topical semisolid product. Also, the flow curves were plotted between the viscosity and shear rate at four distinct temperature levels (i.e., 5, 25, 45, 65 °C) (Figure 1e). The product viscosity exhibited an inverse correlation with the changing temperature: the higher the temperature, the lower the viscosity, and the thinner the product. However, the MTX hydrogel was thermally stable over the shear rate range researched: no substantial change in viscosity was observed when the temperature was modified between 5 and 65 °C. At any temperature, a reduction in the viscosity was obtained with an increasing shear rate. Thixotropic qualities demonstrate structured materials’ capacity to recover after exposure to substantial shear. The structural stability of MTX hydrogel was determined by the oscillatory three-step thixotropy test (Figure 1f). G′ and G″ were observed as two parallel lines during the pre-shear interval, demonstrating the excellent elasticity of the gel structure. Under a high shear rate at 3000 s^−1^, a substantial change in G′ and G″ was observed, showing the abrupt disruption to the structure. During the post-shear interval, the recovery percentage was recorded as 105.85%, while G′ and G″ regained their values and appeared as two parallel lines again, indicating the complete recovery of the gel microstructure. These findings from the thixotropic analysis suggested the stability of the MTX hydrogel, even under significant shear stress.

### 3.2. Ultrastructural Evaluation

The size, geometry, and sharpness of PLGA microneedles, as well as the surface area of microneedle-formed channels in the skin, were investigated employing SEM images of small parts of the microneedle array and skin tissue [8,53]. The microneedle patch contained a central region measuring 4.5 × 4.5 sq·mm and housed 100 solid polymeric microneedles with a rectangular–pyramidal geometry. Ten randomly selected microneedles on an array of each group were examined for the dimensions. Table 1 displays the dimensional parameters, including microneedle length, inter-needle spacing, base size, and tip diameter, of PLGA microneedles. The current study’s findings corroborated our previous studies on PLGA microneedles, where we reported an agreement between the dimensions of the master structure, PDMS mold, and fabricated microneedles [11].

**Figure 2:**
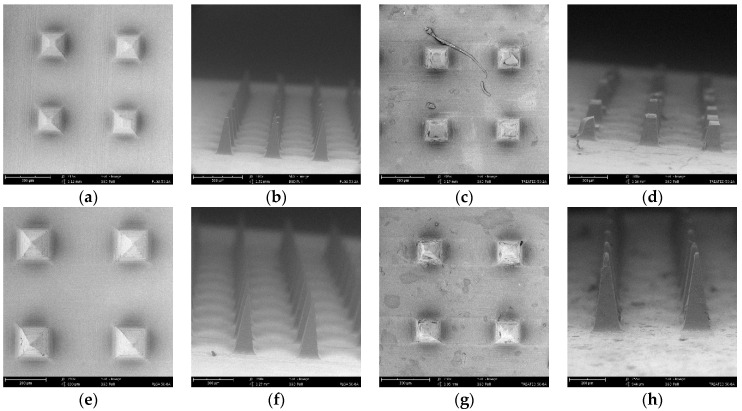
SEM photographs of microneedles: (**a**,**b**) unused and (**c**,**d**) used PLGA 50-2A microneedles; (**e**,**f**) unused and (**g**,**h**) used PLGA 50-8A microneedles. Scale bar: 200 µm.

The analysis of microneedle length and sharpness revealed the significant advantages of PLGA 50-8A microneedles. Microneedles fabricated from PLGA 50-8A exhibited a statistically significant increase in length compared to those prepared from PLGA 50-2A (n = 10, *p* = 0.03), as depicted in Figure 2. Notably, the inter-needle spacing and base dimensions remained statistically comparable between the two types of microneedles (*p* > 0.05). Differently, the average tip diameter of PLGA 50-8A microneedles was markedly lower than PLGA 50-2A microneedles (Table 1). The substantial standard deviation (SD) observed in the tip size of PLGA 50-2A microneedles suggested a dimensional heterogeneity, with some needles possessing sharp, miniature tips, conducive to efficient skin penetration, while others displayed blunt morphologies incapable of inducing significant skin disruption. Microneedles made of PLGA 50-8A were sharp, robust, and consistent in geometry (Figure 2e,f). PLGA 50-8A microneedle arrays mimicked the shape and size of the master structures. These findings endorse the utilization of micromolding as a viable technique for the fabrication of microneedles possessing user-defined geometries. This result aligned excellently with the prior work of Loizidou and coworkers, where a high degree of concordance was observed between the dimensions of PLGA microneedles and the corresponding micromold employed during the production [51]. In line with Miyano et al.’s observations, our PLGA microneedle investigations corroborate the critical role of inter-needle spacing in circumventing the “bed of nail” effect. This phenomenon impedes optimal penetration depth within the skin if the spacing between microneedles, particularly those exceeding 500 μm in length, falls below a threshold of approximately 350 μm [60]. Both PLGA microneedles in the present study were likely to circumvent the skin indentation and penetrate the skin epidermal layer due to their sharpness and length.

After two minutes of insertion into the skin tissue, PLGA 50-2A microneedles exhibited a significant reduction in length and a notable increase in tip dimensions (n = 10, *p* < 0.05). However, the inter-needle spacing and base size remained statistically unchanged. This dimensional alteration in PLGA 50-2A microneedles was most plausibly attributed to physical breakage upon skin insertion, likely due to their fragile mechanical strength, rather than polymer dissolution in the limited interstitial fluid present within the short timeframe (two minutes). Conversely, PLGA 50-8A microneedles displayed dimensional stability following skin insertion. Neither needle length, inter-needle spacing, nor base dimensions exhibited significant alterations (n = 10, *p* > 0.05). However, a statistically significant increase in tip diameter was observed. These findings corroborate prior in vivo studies conducted by Kim and colleagues, who reported no dimensional changes in PLGA microneedles inserted into the backs of hairless mice for one hour [49]. Similarly, Park and coworkers demonstrated effective microneedle application into the skin without fracturing, as indicated by post-insertion microscopic examination [7]. The skin’s moisture content varies between 20% and 60%, depending on the skin layers, and progressively rises from the topmost stratum corneum layer to the epidermis and dermis, leading to increased water absorption near the needles’ tips [61]. Kim and colleagues developed gel microparticle-encapsulated PLGA microneedles. These needles could effectively and sustainably deliver both hydrophilic and hydrophobic compounds into the skin. After the skin insertion, gel microparticles absorbed skin moisture, swelled, and expanded quickly, triggering the mechanical failure of PLGA microneedles [49].

**Figure 3:**
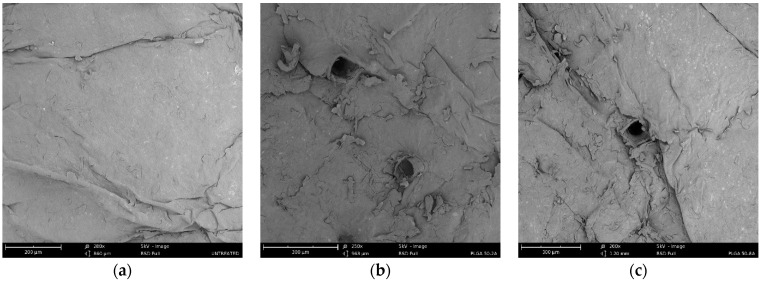
SEM photographs of skin tissue: (**a**) untreated, intact skin, (**b**) skin porated with PLGA 50-2A microneedles, (**c**) skin porated with PLGA 50-8A microneedles. Scale bar: 300 µm.

Scanning electron microscopy (SEM) was employed to delineate the geometric configuration and quantify the surface area of the microchannels generated within the skin. Notably, the observed quadrangular morphology of these channels closely mirrored the geometry of the PLGA microneedles, indicating a direct correlation between microneedle design and resultant microchannel structure (Figure 3b,c). This finding corroborated our earlier research results [11,53,54]. The untreated skin (Figure 3a) and skin region surrounding the channels remained undisturbed by microneedle intervention. Microchannels have been shown to be effective in controlling drug delivery and possible infection [62].

The surface area of channels in human skin generated by PLGA microneedle insertion was accurately measured in this study. Specifically, PLGA 50-2A microneedles yielded microchannels with a surface area of 8567.11 ± 1812.77 sq·μm (n = 10), corresponding to 32.64% of the mean needle base area and 0.92% of the total skin permeation area. Similarly, the insertion of PLGA 50-8A microneedles generated microchannels with a surface area of 9267.40 ± 3519.77 sq·μm (n = 10), representing 34.46% of the mean needle base area and 1.45% of the skin permeation area. These observations were likely attributable to a combination of factors: skin indentation upon microneedle insertion and the skin viscoelasticity likely lead to instant pore contraction upon microneedle removal [53,63,64]. Hence, the microneedle design and construction material would significantly influence the level of skin indentation and microneedle penetration, ultimately impacting drug permeability [65]. Microchannel diameters might vary greatly, depending on the needle shape, measuring methodologies, and application strategies. Prior studies employed confocal laser and fluorescent microscopy to determine channel diameter [54,66] and surface area [53]. However, these methods present potential limitations, including erroneous diameter measurements due to non-circular channel shapes (for diameter measurement), horizontal diffusion of fluorescent dye within the skin tissue, and indistinguishable boundaries of stained channels (to measure the channels’ area using confocal laser microscopy). Notably, the SEM method employed in this study effectively addresses these limitations.

### 3.3. Chemical Characterization

**Figure 4:**
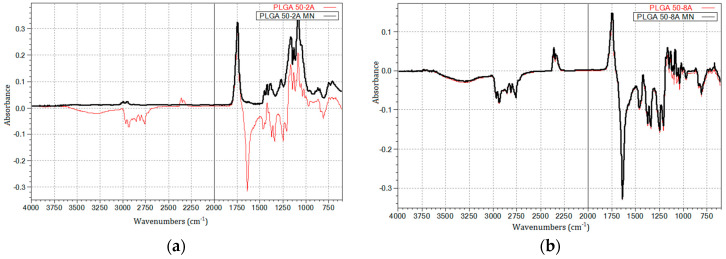
FTIR spectra: (**a**) PLGA 50-2A granules and microneedles, (**b**) PLGA 50-8A granules and microneedles.

Fourier-transform infrared (FTIR) spectroscopy served as a cornerstone technique for elucidating the potential modifications to the chemical structure of PLGA polymers induced by the microneedle fabrication process. FTIR spectra were acquired for both the granules and microneedles of PLGA Expansorb^®^ DLG 50-2A and 50-8A (Figure 4). Notably, a prominent peak, characteristic of both PLGA variants, was observed at 1750 cm^−1^, corresponding to the carbonyl group (–COOH end group) C=O stretching vibration within the two monomers. This reading was consistent with other studies on PLGA [67,68]. The feature peaks of PLGA 50-2A were identified at 1184 and 1089 cm^−1^ (C-O-C stretching) and 1450 cm^−1^ (C-H stretching). The intense peak at 1750 cm^−1^ remained unchanged after exposure to the harsh conditions of the fabrication process (200 mbar vacuum, 180 °C for 30 min) for PLGA 50-2A and 50-8A polymers. The peaks for C-O-C stretching and C-H stretching appeared more prominent in PLGA 50-2A than in PLGA 50-8A. The fabrication process imposed a significant modification to the chemical properties of PLGA 50-2A, especially in C-O-C and C-H stretching (Figure 4a), whereas PLGA 50-8A had no discernible effect (Figure 4b).

### 3.4. Mechanical Analysis

**Figure 5:**
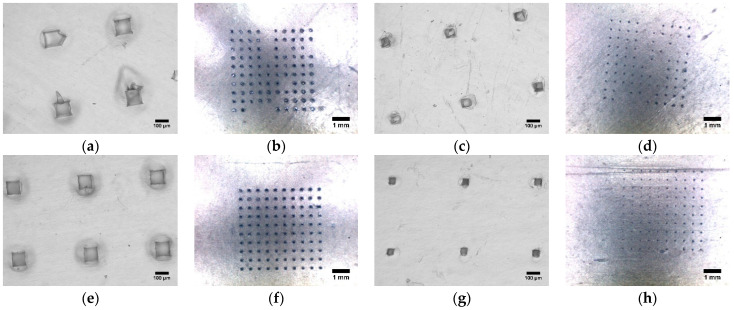
Microscopic photographs of Parafilm M^®^ layers porated with PLGA microneedles: (**a**,**b**) first layer, (**c**,**d**) second layer for PLGA 50-2A microneedles; (**e**,**f**) first layer, (**g**,**h**) second layer for PLGA 50-8A microneedles.

Parafilm served as a platform to assess the mechanical robustness and uniformity of the fabricated PLGA microneedles. We observed a series of well-defined quadrate channels on the parafilm layers, mirroring the pyramidal geometry of the microneedles. The uppermost parafilm layer exhibited a significantly greater channel area for PLGA 50-8A microneedles compared to PLGA 50-2A microneedles (*p* < 0.05). This trend persisted on the second layer, signifying a statistically significant reduction in pore area for both microneedle types as the needles traversed deeper into the parafilm (*p* < 0.05, Figure 5). The modest deviations in the area of the pores demonstrated that the pores were comparable in size, indicating the mechanical consistency of PLGA microneedles. Intriguingly, the pore area on all parafilm layers remained considerably smaller than the microneedle base size (*p* < 0.05). This suggests incomplete penetration of PLGA microneedles into parafilm layers, or part of the needle length could enter the parafilm effectively. The inter-pore spacing on the first layer displayed negligible disparity between PLGA 50-2A (519.56 ± 4.93 μm, n = 10) and PLGA 50-8A microneedles (521.59 ± 4.24 μm, n = 10, *p* = 0.34). Moreover, a distinct, circular indentation surrounding each pore corroborated the microneedle-induced deformation of the parafilm layers (Figure 5a,e). The presence of 100 channels on the second layer porated with PLGA 50-8A needles signified the excellent uniformity in the microneedles’ length, enabling consistent penetration across the entire flat and homogenous parafilm surface. This finding translated to a remarkable 100% penetration efficiency for PLGA 50-8A needles, aligning with our prior investigation utilizing Parafilm M^®^ to assess PLGA microneedle uniformity [11]. Conversely, the inability to locate 100 channels on the last parafilm layer porated by PLGA 50-2A needles translated to a significantly lower penetration efficiency (91.30 ± 3.20%; n = 10, *p* < 0.05) compared to PLGA 50-8A microneedles.

In a previous study, we studied the mechanical robustness of PLGA microneedles subjected to compressive and shear forces (axial and transverse loadings). Under increasing axial pressure, PLGA microneedles bent unilaterally and resisted failure from fracture. Disruption of a linear array of ten PLGA microneedles necessitated a transverse force of 1.61 N [11]. Loizidou and colleagues revealed that microneedle geometry substantially impacted their mechanical strength. The mechanical integrity of the microneedles exhibited a positive correlation with the complexity of the polygonal structure, as evidenced by their enhanced capacity to withstand increasing compressive loads. Hexagonal-shaped microneedles, in particular, were less likely to break than triangular microneedles. Microneedle geometries with a triangular-shaped base exhibited a demonstrably greater propensity for buckling failure compared to alternative designs [51]. Furthermore, the intrinsic mechanical robustness of the polymeric microneedle was directly contingent upon the material used to construct the polymer matrix. Materials characterized by a greater Young’s modulus produced microneedles with demonstrably enhanced mechanical strength, facilitating superior penetration efficiency [69,70]. The Young’s modulus of PLGA (50 kDa) was determined to be around 1 Gpa [71]. Therefore, the mechanical robustness of PLGA needles was determined by the composition, molecular weight, and Young’s modulus of PLGA polymer. Park and coworkers evaluated the failing force of PLGA microneedles, with varying lengths, fixed tip dimensions, and base size. As a result, the failure force reduced as the needle length increased (*p* < 0.001) [7]. The observed inverse correlation between critical load and column height was likely attributable to a reduction in the buckling threshold with increasing column height. David et al. revealed that the microneedle insertion force depended primarily on the interfacial area at the tip rather than the other geometric factors [72]. Thus, the dimensions and structure of the microneedle tips are of crucial importance for their penetration efficiency.

### 3.5. Dye Binding Studies

**Figure 6:**
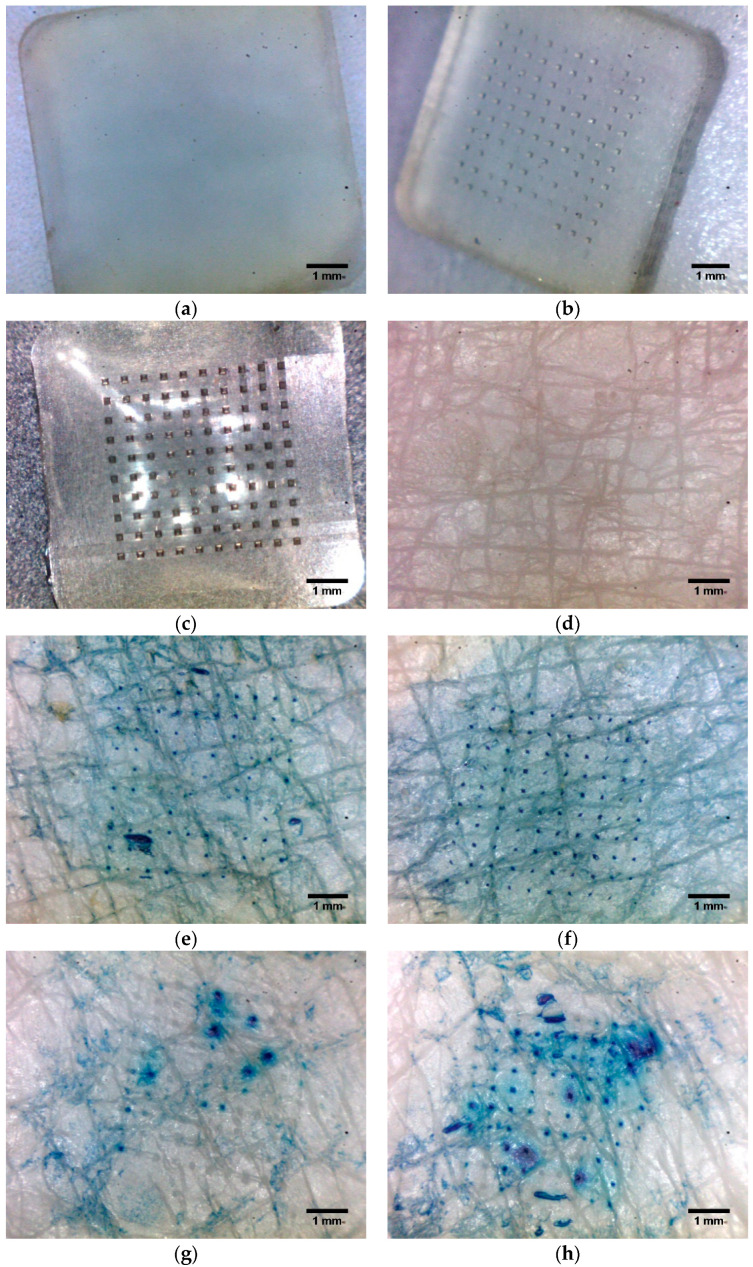
Microscopic photographs of microneedles and skin: (**a**) base substrate, (**b**) PLGA 50-2A microneedles, (**c**) PLGA 50-8A microneedles, (**d**) untreated human skin, skin porated with PLGA 50-2A microneedles employing (**e**) a spring-loaded applicator, (**g**) thumb pressure, and skin porated with PLGA 50-8A microneedles employing (**f**) a spring-loaded applicator, (**h**) thumb pressure.

Dye binding experiments were performed to assess the insertion efficiency of PLGA microneedles into human skin in vitro. The untreated, intact skin remained unperturbed by the methylene blue solution. This impermeability can be attributed to the inherent hydrophilicity of the dye, which impedes its passage through the intact stratum corneum, a naturally hydrophobic barrier. For microneedle-treated skin tissues, the channels were filled with skin interstitial fluid, thus easily absorbing the blue dye, resulting in conspicuous staining, while the surrounding areas remained undisrupted and did not uptake the dye (Figure 6a–d). This observation demonstrated an effective skin microporation generated by the insertion of both microneedle types. Our findings concurred with prior investigations, which demonstrated that methylene blue selectively permeated microchannels created in the skin, whereas the vicinity remained impermeable to the dye [8,53,54]. The microchannels, visualized as blue-stained regions, were dispersed consistently with the needle pattern on the array. Inter-needle and inter-pore spacing analysis revealed a statistically insignificant difference between PLGA 50-2A and PLGA 50-8A microneedles. This observation also held true for inter-pore spacing on the parafilm layer (n = 10 for each microneedle group, *p* > 0.05). Our previous study on PLGA microneedles also found that 100 solid PLGA microneedles successfully porated porcine ear skin, creating 100 uniformly distributed channels in the skin tissue. The inter-pore spacing on the skin was comparable to the inter-needle spacing on the microneedle patch [11]. Likewise, PLGA 50-8A microneedles outperformed their PLGA 50-2A counterparts in human skin microporation. As depicted in Figure 6f, PLGA 50-8A microneedles effectively generated a robust network of 100 microchannels. Conversely, PLGA 50-2A microneedles yielded a significantly lower number of channels (68.50 ± 12.89, n = 10). This disparity was further accentuated by the observation that PLGA 50-2A microneedles created a considerably higher number of channels on parafilm layers (91.30 ± 3.20%). This result was attributable to the noticeable viscoelasticity of skin that imposed certain physical resistance and indentation upon microneedle insertion. This physical resistance of skin tissue was more prominent than parafilm layers. Furthermore, the utilization of a spring-loaded applicator yielded a greater penetration efficiency when juxtaposed with manual thumb pressure (Figure 6). The applicator applied consistent pressure to each microneedle, resulting in more homogenous microchannels in the skin. The preliminary comparison between thumb pressure and spring-loaded applicator was performed only in this dye binding study. Similarly, other studies have revealed that the use of an applicator may drastically improve penetration, playing a critical role in the pressure and speed of microneedle insertion [73,74,75]. In another experiment, Kim and coworkers fabricated gel particle-incorporated PLGA microneedles and reported that the microneedle insertion depended heavily on the mechanical robustness and composition of the PLGA polymer. In particular, microneedles with 53% (*v*/*v*) gel particles could be inserted into the skin successfully (100% microneedles penetrated skin), whereas, for microneedles with 68% gel particles, only approximately 75% of the number of microneedles on the array could disrupt skin tissue to successfully create microchannels [49].

### 3.6. Histological Analysis

**Figure 7:**
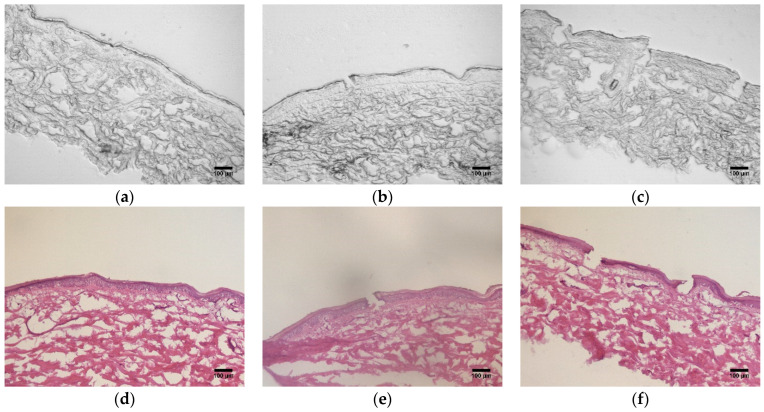
Histological examination of skin tissue: (**a**,**d**) untreated, (**b**,**e**) treated with PLGA 50-2A microneedles, and (**c**,**f**) treated with PLGA 50-8A microneedles. (**a**–**c**) Unstained tissues, (**d**–**f**) skin tissues stained with hematoxylin and eosin.

The histological analysis of cryosectioned skin tissue was performed to confirm the creation of microchannels by microneedle application [53,66]. As shown in Figure 7, the microscopic evaluation of the cross-sections revealed distinguishable skin strata with distinct colors. While skin tissue appeared intact and unaffected in the untreated group, we observed a significant disruption in microneedle-treated skin. This finding demonstrated the effectiveness of microneedle insertion in penetrating the epidermal layer to enter the upper dermal layer (Figure 7). Furthermore, the channels’ depth was estimated at around 120 µm for both needle types. Historically, histological analysis served as the primary means to assess the insertion depth of microneedles. This process involved fixing, freezing, sectioning, and staining microneedle-treated skin tissue [64,76]. This cumbersome histological sectioning carried certain risks of errors. The physical distortion of skin could substantially modify the channels’ dimensions. Accurate pore depth determination necessitated cryosection collection precisely at the channel’s center, introducing potential sampling bias. Furthermore, the viscoelasticity of skin tissue rendered microchannels susceptible to dimensional changes upon microneedle removal. To overcome these drawbacks, advanced technologies have been implemented to precisely measure the depth of microneedle-created microchannels, such as confocal laser scanning microscopy [54,70,77] or optical coherence tomography [73,78,79].

### 3.7. Microchannel Uniformity

**Figure 8:**
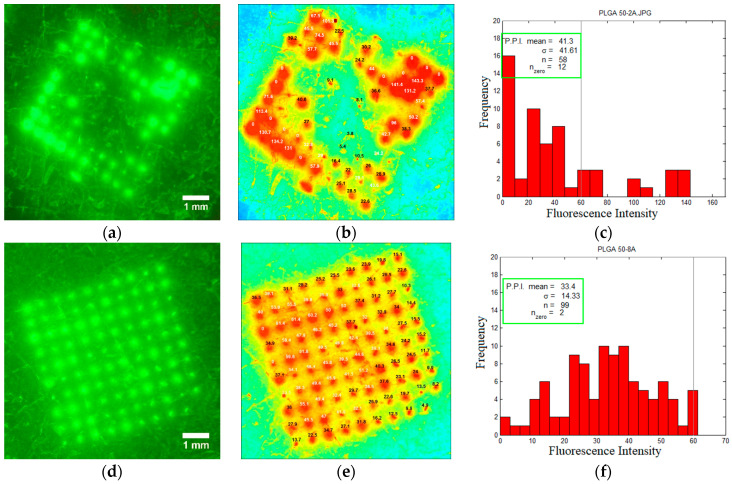
Fluorescent photographs with Pore Permeability Index and distribution of fluorescence intensity of skin: (**a**–**c**) skin porated with PLGA 50-2A microneedles, (**d**–**f**) skin porated with PLGA 50-8A microneedles.

High-resolution fluorescence photographs were captured using a digital camera with dedicated macrolens and a long-pass filter tuned to 525 nm. Subsequent image analysis, facilitated by Fluoropore software, enabled the quantification of fluorescent intensity distribution across individual microchannels. This distribution served as an indicator for the relative flux of calcein within each channel [2]. Consequently, a bell-shaped histogram depicting the fluorescence intensity distribution (Figure 8f) corroborated the exceptional uniformity of microchannels generated by PLGA 50-8A microneedles. Furthermore, we employed the Pore Permeability Index (PPI) to quantify microchannel uniformity. This analysis revealed a statistically significant difference between the two microneedle types (*p* < 0.05). Microchannels generated by PLGA 50-2A microneedles exhibited a higher standard deviation in PPI values (41.30 ± 41.61, n = 58, n_zero_ = 12), indicative of less uniformity, compared to those generated by PLGA 50-8A microneedles (33.4 ± 14.33, n = 99, n_zero_ = 2) (Figure 8). These findings demonstrated the superior efficacy of PLGA 50-8A microneedles in generating more uniform channels within human skin than PLGA 50-2A microneedles.

### 3.8. Microchannel Depth

**Figure 9:**
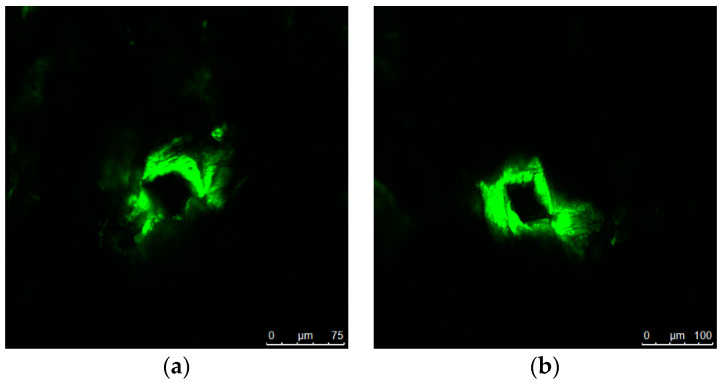
Confocal microscopic photographs of skin: (**a**) treated with PLGA 50-2A microneedles, (**b**) treated with PLGA 50-8A microneedles.

**Figure 10:**
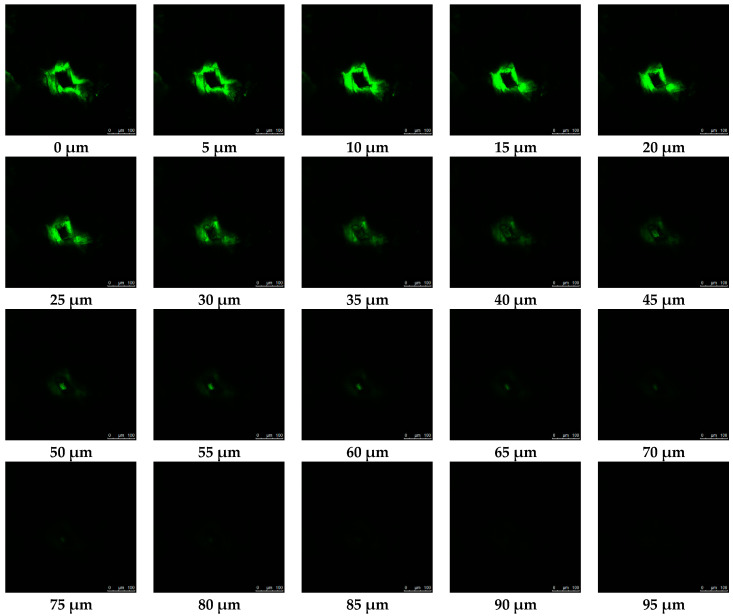
Confocal z-stack of microchannel generated by the insertion of PLGA 50-8A microneedles.

The depth of effective microneedle penetration is determined by several factors, such as duration, velocity, pressure, type of insertion technique, dimensions and material of construction of microneedles, angle of microneedle insertion, viscoelasticity, and surface properties of skin tissue. Among those, the most critical attributes were needle length and inter-needle spacing [80,81], tip diameter, base dimensions [82,83,84], and material of construction [7,69,70]. Confocal laser scanning microscopy has emerged as a well-established technique for precisely quantifying the depth of microchannels generated by microneedles within the skin. This non-invasive and expeditious method leverages the selective infiltration of hydrophilic calcein dye into the disrupted regions, specifically the microchannels. Conversely, the surrounding intact stratum corneum, characterized by its hydrophobic properties, remains unstained by the dye, enabling a clear demarcation of the microchannel dimensions. Consequently, the fluorescence intensity of calcein served as a precise cartographic representation of the microchannels’ location, geometry, and depth. The dye was used to stain the microchannels, not the surrounding regions, to make them noticeable on confocal fluorescence images (Figure 9a,b). In addition, we observed no calcein staining in hair follicles, which were invisible in confocal images. The large molecular weight of this hydrophilic dye hindered its diffusion into the follicular pathway. Furthermore, a multi-planar z-scan was employed to elucidate the three-dimensional distribution of the fluorescent dye within the microchannels. This technique involved capturing a series of high-resolution microscopic images at progressively deeper depths (z-axis) within the skin, maintaining a constant horizontal position (x, y). Image acquisition commenced at the skin’s surface, where the fluorescent signal exhibited its greatest intensity and areal extent. This process continued until the signal became indiscernible, signifying either the termination of the microchannel or the absence of the fluorescent dye (Figure 10) [11,53]. This z-scan generated a sequence of confocal photographs with a progressive decline in fluorescent intensity proportional to the depth of the channels. Following calculation via multiplication of z-step number and step size (5 µm), the channel depths generated by PLGA 50-2A microneedles (74.00 ± 23.90 µm, constituting 18.83% of microneedle length) were markedly shallower than those formed by PLGA 50-8A microneedles (99.00 ± 7.75 µm, representing 22.05% of microneedle length, n = 10, *p* < 0.05). As presented in Table 1, this observation underscored the principle that pore depth remained consistently inferior to microneedle length. Furthermore, a positive correlation was observed between increased needle length and the resulting depth of the formed channels. This result could be attributed to the viscoelasticity and indentation of skin upon microneedle insertion. Other investigations have demonstrated a correlation between insertion depth, microneedle length, and inter-needle spacing. The “bed-of-nails” effect negatively impacted insertion efficiency when the spacing between needles was smaller than 150 µm [73,74,85]. The inter-needle spacing in the present study (approximately 520 µm) had a large safety margin over this threshold; thus, we minimized the bed-of-nails effect in our PLGA microneedles. The depths of microchannels generated by PLGA 50-2A and 50-8A microneedles were unable to disrupt the dermis layer where blood capillaries and nerve fibers are located to create bleeding, irritation, or pain. Loizidou and colleagues evaluated the insertion efficiency of PLGA microneedles possessing various geometric configurations, including triangular, square, and hexagonal base designs. Their investigation revealed that the square-shaped microneedles achieved the greatest penetration depth, reaching a mean distance of 657 µm (34% penetration) from the microneedle substrate to the skin surface. Triangular microneedles exhibited comparable penetration efficiency (660 µm, 34% penetration), while hexagonal geometries demonstrated a shallower penetration depth of 803 µm (20% penetration). Increased mechanical strength of microneedles was achieved with greater numbers of vertices in a polygonal structure but decreased their skin penetration efficiency. Comparative evaluations revealed that hexagonal microneedles exhibited superior resilience to compressive force and critical buckling loads compared to their square and triangular counterparts. However, a shallower penetration depth accompanied this enhanced mechanical robustness [51]. Lee et al. showed that pyramidal-shaped needles with square or triangular base geometries penetrate the skin to a depth of one-third of the needle length [69], while hexagonal-shaped microneedles provide a penetration depth of 20% microneedle length. Loizidou et al. concluded that square pyramidal microneedles—the design and geometry of PLGA microneedles in the present study—offered an excellent balance between insertion efficiency and mechanical robustness [51].

### 3.9. Skin Integrity Evaluation

**Figure 11:**
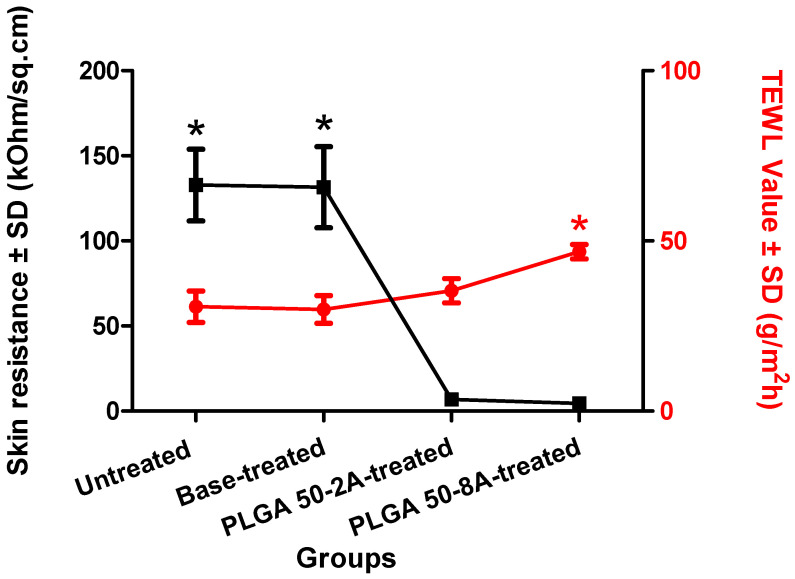
Transepidermal water loss and skin electrical resistance values of untreated (control), base-treated, and PLGA microneedle-porated skin (* denotes statistically significant difference from the untreated and base-treated groups, mean ± SD, n = 4, *p* < 0.05).

In vitro skin integrity assessment employed transepidermal water loss (TEWL) and skin electrical resistance measurements. A compromised skin barrier permits increased water evaporation and elevated TEWL values [2]. Meanwhile, this disruption would create a pathway to facilitate ion passage across the skin, thereby causing a significant and sudden reduction in skin electrical resistance [11]. In this study, we reported a marked drop in skin resistance upon microneedle insertion (Figure 11, n = 4, *p* < 0.01). However, no significant difference in resistance was evident between the untreated and base-treated groups (*p* = 0.94), nor between the two microneedle groups (*p* = 0.06). This finding revealed that the application pressure of a base substrate on skin provided no modification to skin integrity. Similarly, the microneedle-created physical disruption to the skin was completely caused by the needles, supported by no impact from the array base. Furthermore, skin resistance measurement was unable to distinguish the significantly different levels of skin disruption caused by PLGA 50-2A and 50-8A microneedles. There was no relationship between the total channel area and the electrical resistance of the treated skin, which fell significantly to a confined range. The decrease in skin electrical resistance was previously reported by Sivaraman and coworkers, as the application of maltose microneedles resulted in a 10% reduction in the electrical resistance of dermatomed human skin [86].

Remarkably, the TEWL value of skin treated by PLGA 50-8A microneedles (46.85 ± 2.14 g/m^2^h) was significantly greater than that of untreated skin (30.68 ± 4.50 g/m^2^h), base-treated skin (29.88 ± 4.11 g/m^2^h), and those porated by PLGA 50-2A microneedles (35.40 ± 3.57 g/m^2^h) (n = 4, *p* < 0.05) (Figure 11). These findings indicated the effective skin microporation by the PLGA needle insertion. Furthermore, the TEWL value appeared sensitive to the level of skin disruption in different treatment groups. Our investigation revealed a direct correlation between the total surface area of microneedle-formed channels and the TEWL value, which serves as a marker for the extent of skin poration. The observed increase in total channel area associated with the transition from PLGA 50-2A to PLGA 50-8A microneedles translated to a statistically significant elevation in TEWL. This finding aligns with prior reports, demonstrating a similar increase in TEWL values caused by microneedle-mediated skin microporation [2,11,13]. For instance, Sivaraman et al. observed a rise in TEWL values (14.1 to 21.2 g/m^2^h) following the application of maltose microneedles, indicating that the needles disturbed the stratum corneum [86]. Similarly, Vemulapalli et al. reported an increment in the TEWL values from 6.2 to 10.4 g/m^2^h after microneedle insertion and increased from 7.2 to 10.64 g/m^2^h when combined with iontophoresis [16].

### 3.10. Permeation Studies

**Figure 12:**
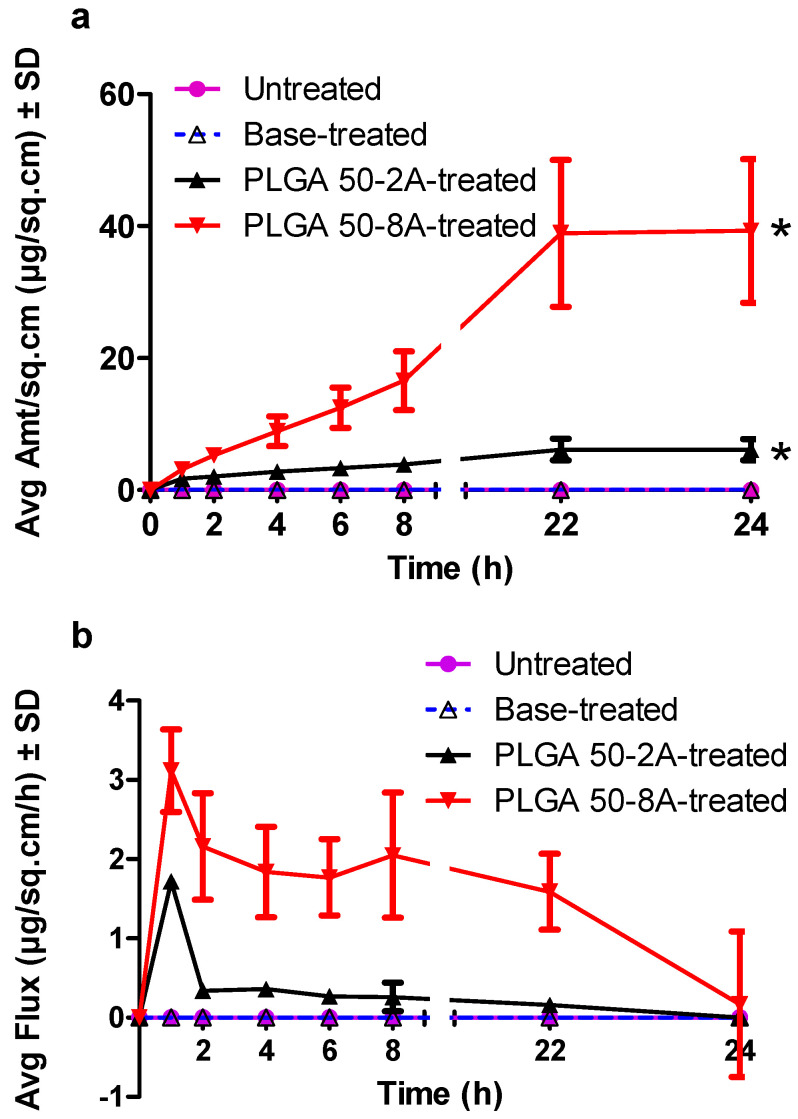
In vitro transdermal permeation of methotrexate: (**a**) average cumulative quantity and (**b**) transdermal flux of methotrexate delivered across untreated, base-treated, and PLGA microneedle-porated skin (* denotes statistically significant difference from the untreated and base-treated groups, mean ± SD, n = 4, *p* < 0.05).

In this experiment, we employed Franz vertical diffusion cells to quantify methotrexate (MTX) permeation into and across untreated (control), base-treated, and PLGA microneedle-porated skin in vitro. Methotrexate exhibited negligible permeation (0.00 ± 0.00 µg/sq·cm) across the intact stratum corneum in the untreated (control) group. This impermeability can be attributed to the inherent hydrophilicity of MTX with a log *p* value of −1.85, moderate molecular weight, and ionized propensity at bodily pH [87] (Figure 12). This finding necessitated the employment of enhancement technologies to improve the transdermal drug delivery. Likewise, Lee et al. reported a negligible absorption of MTX by passive diffusion [25]. Our previous research on MTX transdermal delivery assisted by PLGA microneedles found the same result of no MTX passing through untreated porcine ear skin in vitro by passive diffusion [11]. Similarly, Vora and colleagues revealed no passive diffusion of MTX through healthy human skin [13]. Nevertheless, in a prolonged 72 h in vitro permeation experiment, Sivaraman and colleagues observed a considerable disparity in methotrexate delivery across formulations. In particular, 0.2% *w*/*w* MTX poloxamer-containing formulations drove a significantly lower quantity of methotrexate into the receptor solution compared to their non-poloxamer counterparts (*p* < 0.05). This trend persisted at the higher concentration (0.4% *w*/*w*), with poloxamer formulations facilitating a modest delivery of 4.15 ± 1.20 μg/sq·cm, while non-poloxamer formulations yielded a substantially higher amount (60.94 ± 48.81 μg/sq·cm) [86]. Such a passive permeation could be attributed to the extended study timeframe (skin tissues became hydrated and swellable, thus compromising the skin integrity and barrier function), greater MTX content, and different drug formulations.

We observed no enhancement in MTX delivery after the insertion of the array base substrate. This finding revealed that the array substrate had no detrimental effect on the skin barrier function. Also, any observed disruption to the skin integrity was solely attributable to the transient physical interaction with PLGA microneedles. Microneedle design emerged as a critical determinant of controlled drug delivery, independent of the array substrate. MTX delivery was significantly improved by PLGA microneedle application (*p* < 0.05) (Figure 12). Our previous work demonstrated that microneedle insertion altered skin rheology—the skin’s physical structure became loosened and exhibited reduced elasticity [53]. Previously, we reported that PLGA microneedles significantly enhanced MTX permeation through porcine ear and dermatomed human cadaver skin in vitro when compared to the untreated group (n = 4, *p* < 0.05) [11]. Panda et al. fabricated dissolving microneedles from PLGA 50:50 and PLGA 65:35 and reported a faster release of FITC-dextran from PLGA 50:50 microneedles than PLGA 65:35 microneedles due to a greater glycolic acid content [50]. Vora et al. revealed that the skin treatment with Dr. Pen^TM^ Ultima A6 led to significantly higher drug delivery across full-thickness human skin compared to the untreated (passive permeation) group. Furthermore, the combination of microneedles and iontophoresis delivered a markedly greater quantity of drug through the skin than microneedles or iontophoresis alone [13]. Sivaraman et al. demonstrated a substantial increase in transdermal MTX delivery upon the application of solid maltose microneedles compared to passive diffusion. This enhancement was observed for both poloxamer and non-poloxamer formulations at MTX concentrations of 0.2% *w*/*w* and 0.4% *w*/*w* [86]. Likewise, a 20-needle array enhanced the skin permeability of calcein and bovine serum albumin by at least twofold, but a 100-needle patch improved the drug delivery by about threefold [7]. In in vivo research, Vemulapalli and coworkers discovered that the application of soluble maltose microneedles created multiple channels in the skin and substantially enhanced MTX transdermal delivery in hairless rats [16]. A comprehensive review was published by Shah et al. to present the high demand for minimally invasive and microneedle-based transdermal drug delivery systems to efficiently deliver MTX across skin [88]. We also reported a significantly greater quantity of drug entering the receptor chamber by PLGA 50-8A microneedles compared to their PLGA 50-2A counterparts (n = 4, *p* < 0.01). The enhancement in MTX transdermal delivery by PLGA 50-8A microneedles likely stems from their superior capacity to create a significantly greater number and depth of microchannels within the skin, as demonstrated in dye binding and confocal analysis. These findings suggest a positive correlation between the microneedle’s penetration efficiency and the efficacy of transdermal MTX delivery.

Analysis of lag times revealed a statistically significant difference between PLGA 50-2A (4.16 ± 0.74 h) and PLGA 50-8A microneedles (1.33 ± 0.42 h) (n = 4, *p* < 0.01, Table 2). These results indicated that PLGA 50-8A microneedles effectively expedited the onset of MTX permeation. This was attributable to the deeper microchannels generated by PLGA 50-8A microneedles, which facilitated the placement of MTX within the dermis, thereby reducing the tortuosity of the drug permeation path and, consequently, accelerating the drug delivery. Interestingly, a reduction in lag time has been identified as a critical factor for pediatric drug delivery [2]. Furthermore, the physical disruption of the skin barrier by PLGA microneedles led to a considerable improvement in the transdermal flux, diffusion coefficient, and permeability coefficient (*p* < 0.05, Table 2). These findings collectively suggest the superiority of PLGA 50-8A microneedles compared to PLGA 50-2A microneedles in enhancing transdermal drug delivery. A drop in the lag time of MTX delivery caused by microneedle treatment was also reported elsewhere [11,13]. A single array of microneedles, comprising 100 PLGA 50-8A microneedles generating channels of 0.0093 sq·cm total area, facilitated the transport of methotrexate at a flux of 1.69 ± 0.54 µg/sq·cm/h; an array consisting of 69 PLGA 50-2A microneedle forming microchannels of 0.0059 sq·cm total area provided a flux of 0.34 ± 0.05 µg/sq·cm/h. Extrapolating these findings to a hypothetical 1 sq·cm area densely packed with microchannels, PLGA 50-8A microneedles were projected to generate 10,791 channels compared to 11,673 for PLGA 50-2A microneedles. This translated to a significant enhancement in predicted flux, reaching 182.37 µg/sq·cm/h for PLGA 50-8A compared to 57.52 µg/sq·cm/h for PLGA 50-2A microneedles.

The present study’s employment of human skin resulted in more practical and relevant insights for clinical trials involving human volunteers. Similarly, Lee and colleagues affirmed that human skin offers the most dependable database for cutaneous absorption [25]. Employing a clearance rate of 118 mL/h for MTX [89], we used Equation (6) to calculate the steady-state plasma concentrations of 311.58 ± 45.23 ng/mL (PLGA 50-2A microneedles) and 988.92 ± 294.85 ng/mL (PLGA 50-8A microneedles) (Table 2). These values exceeded the targeted plasma concentration of 337 ng/mL reported in 70 kg adult subjects, typically achieved through oral dosing. Further calculations indicated that microneedle array sizes of 1.10 ± 0.17 sq·cm (PLGA 50-2A microneedles) and 0.36 ± 0.11 sq·cm (PLGA 50-8A microneedles) would be necessary to attain the targeted plasma concentration of MTX. Previous in vivo investigations utilizing hairless rats, as reported by Vemulapalli and coworkers, suggested that a maltose microneedle array of 32.5 sq·cm in surface area would be necessary to achieve comparable plasma concentrations to those observed following oral administration [16]. Abla and colleagues predicted that a microneedle array of 13 sq·cm area housing 845 needles would deliver therapeutic levels of MTX [2]. To achieve a therapeutic dose of 22.51 mg of methotrexate for dermatomyositis, a microneedle array encompassing a surface area of 20 sq·cm and incorporating approximately 1267 microneedles would be required [2,90]. Interestingly, D. Vora et al. predicted a transdermal delivery of 2.14 mg/day to achieve the equivalent plasma concentration to a 25 mg once-a-week oral dose. Furthermore, the desired daily drug delivery was estimated to be 42.8 µg/sq·cm [13]. Prior research by Kumar and colleagues revealed the efficacy of a 0.25% MTX gel for treating psoriatic plaques, achieving plaque clearance in 82% of subjects. The hydrophilicity of the gel hindered its penetration into the thick, hyperkeratotic psoriatic regions on palms and soles. Also, the 0.25% MTX concentration was insufficient to treat these areas [27]. This claim agreed with the results of our study, where the gel formulation with a low drug content (0.1%) could deliver no drug across the intact skin by passive diffusion. Generally, the low passive permeation of MTX necessitates greater drug content in formulations, which can, unfortunately, lead to adverse effects, such as burning, erythema, and blistering [91,92]. In the present work, we employed minimally invasive microneedle treatment in tandem with low drug concentration in the hydrogel (0.1% *w*/*w*). This strategy offered the potential to mitigate side effects, thereby promoting patient tolerability, acceptability, and compliance.

### 3.11. Skin Disposition Studies

**Figure 13:**
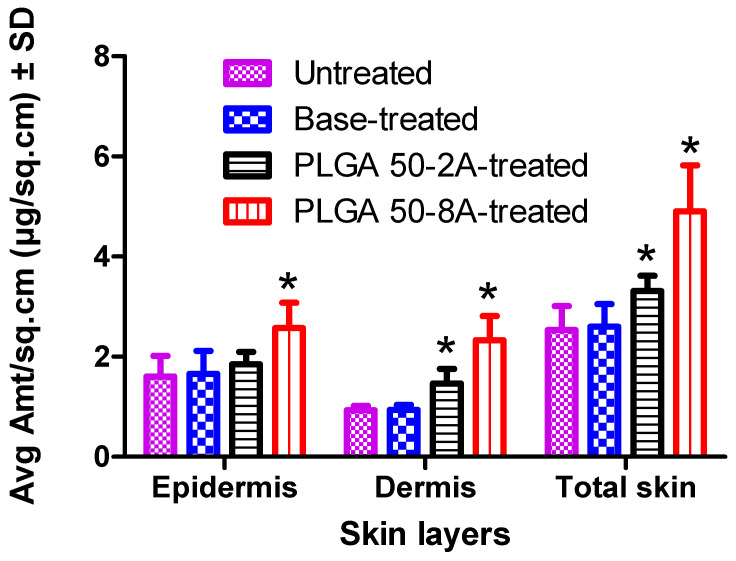
Quantities of methotrexate in skin tissue: untreated, base-treated, and PLGA microneedle-porated skin (* denotes statistically significant difference from the untreated and base-treated groups, mean ± SD, n = 4, *p* < 0.05).

Following in vitro permeation studies, we delineated the biodistribution and quantified the intratissue concentrations of methotrexate (MTX) within distinct epidermal and dermal compartments. In line with the permeation data, PLGA 50-8A microneedles markedly enhanced MTX delivery into the epidermis, dermis, and total skin compared to untreated (control), base-treated, and PLGA 50-2A microneedle groups (*p* < 0.05, Figure 13). The untreated group provided a significantly lower MTX quantity in skin tissue compared to both PLGA 50-2A and PLGA 50-8A microneedle groups (n = 4, *p* < 0.05). No statistically marked differences in MTX quantities were reported between the control and base-treated groups across the epidermis, dermis, and total skin (*p* > 0.05). The efficacy of microneedle application exhibited consistent patterns within the epidermal and dermal layers: both PLGA types significantly increased MTX amounts within both compartments of human skin. Previous research showed that PLGA microneedle microporation significantly increased MTX delivery into porcine ear skin (n = 4, *p* < 0.01). Furthermore, this observation was also reported in microneedle-porated dermatomed human cadaver skin. However, quantitative analysis revealed no statistically significant difference in drug quantities within the stratum corneum layer of porcine ear skin tissues porated with microneedles compared to the untreated control group [11].

We observed no marked difference in the total drug delivery between the control and base-treated groups (n = 4, *p* = 0.84, Table 2). Notably, a markedly higher total delivery in PLGA 50-2A and 50-8A microneedle groups than in untreated and base-treated groups demonstrated the enhanced transdermal drug delivery induced by PLGA microneedle insertion (*p* < 0.01). Microneedles fabricated from PLGA 50-8A facilitated a significantly greater cumulative quantity of drug deposition within and traversing human skin than PLGA 50-2A microneedles (n = 4, *p* < 0.01). Since MTX quantities applied on the skin surface (100 µL MTX hydrogel 0.1% *w*/*w* or 100 µg MTX added to the donor compartment) were comparable among the groups, the difference in the delivery efficiency followed a similar pattern as the total drug delivery. Interestingly, we calculated and reported the topical selectivity of untreated, base-treated, and microneedle-treated groups. No physical treatment or base compression resulted in 100% drug delivered into the skin layers. This observation indicates that all drugs were poorly delivered and trapped in the skin layers following passive permeation. PLGA 50-2A microneedles (35.86 ± 7.37%) delivered a markedly greater proportion of the drug into the skin tissue than PLGA 50-8A microneedles (11.60 ± 3.36%, *p* < 0.05), which drove the majority of MTX across the skin and into the receptor chamber. This finding further validates the superior performance of PLGA 50-8A for enhanced transdermal delivery of MTX. PLGA 50-8A microneedles generated 100 channels (mean depth of 99.00 ± 7.75 µm) on the skin to place the drug onto the deeper layers of the skin (i.e., dermis layer), leaving markedly less drug on the skin surface.

## 4. Conclusions

In the present investigation, we leveraged biodegradable polymeric microneedles to strategically disturb the stratum corneum, the skin’s primary permeability barrier, and establish a network of microchannels to facilitate enhanced transdermal transport of methotrexate across human skin in vitro. PLGA Expansorb^®^ DLG 50-2A and DLG 50-8A microneedles were prepared via the micromolding method and comprehensively evaluated using a battery of techniques, including scanning electron microscopy, Fourier-transform infrared spectroscopy, and mechanical testing. A methotrexate-containing hydrogel formulation was developed and evaluated for critical parameters, such as pH, density, drug assay, content uniformity, and rheological properties. The efficacy of microneedle-mediated skin microporation was demonstrably confirmed through dye binding, histological examination, and the evaluation of skin integrity. Furthermore, the dimensions (surface area and depth) of the microchannels within the skin were quantified using scanning electron microscopy, pore uniformity analysis, and confocal microscopy. Human skin and Franz diffusion apparatus were employed to assess in vitro drug permeation into the receptor compartment and skin strata.

Notably, a statistically marked difference in microneedle length was observed between PLGA 50-8A and PLGA 50-2A microneedles, while the dimensional parameters of the microchannels formed in the skin exhibited no marked disparity. The failure in the skin integrity by the microneedles resulted in a considerably decreased electrical resistance and a significant increment in transepidermal water loss, both of which were indicative of compromised skin integrity. Moreover, the results of the permeation experiments indicated that the application of PLGA 50-8A microneedles yielded a significantly higher drug permeation than the PLGA 50-2A microneedles, base-treated, and control groups (n = 4, *p* < 0.05). Additional parameters evaluated and compared across the groups included lag time, flux, diffusion coefficient, permeability coefficient, and predicted steady-state plasma concentration of the drug. Notably, the application of PLGA 50-8A microneedles led to a substantially greater quantity of methotrexate delivered into the skin tissue than PLGA 50-2A microneedles. Conclusively, this study provides compelling evidence that microneedle treatment offers a viable strategy for significantly enhancing the transdermal delivery of MTX in vitro.

## Figures and Tables

**Table 1:** Dimensional parameters of fabricated PLGA microneedles (n = 10).

Microneedles	Needle Length (µm)	Needle-to-Needle Distance (µm)	Base Side (µm)	Tip Diameter (µm)	Pore Area in Skin (sq·µm)	Pore Depth (µm)
PLGA 50-2A	393.46 ± 74.89	518.36 ± 8.30	162.20 ± 6.88	21.62 ± 17.98	NA ^a^	NA ^a^
PLGA 50-8A	449.82 ± 9.94	516.63 ± 6.39	164.03 ± 4.73	3.66 ± 1.09	NA ^a^	NA ^a^
Treated PLGA 50-2A	161.77 ± 65.94	516.92 ± 11.64	163.88 ± 11.99	104.61 ± 32.24	8567.11 ± 1812.77	74.00 ± 23.90
Treated PLGA 50-8A	446.54 ± 7.11	515.30 ± 10.02	162.08 ± 9.36	16.65 ±2.19	9267.40 ± 3519.77	99.00 ± 7.75

^a^ Not applicable.

**Table 2:** In vitro permeation parameters (transdermal delivery, lag time, flux, diffusion coefficient, permeability coefficient, steady-state plasma concentration, total delivery, delivery efficiency, and topical selectivity) of methotrexate delivered through human skin (n = 4).

Group	Q_24_ ^a^ (µg/sq·cm)	T_lag_ ^b^ (h)	J_ss_ ^c^ (µg/sq·cm/h)	D ^d^ × 10^−5^ (sq·cm/h)	K_p_ ^e^ × 10^−4^ (cm/h)	C_ss_ ^f^ (µg/L)	Total Delivery ^g^ (µg/sq·cm)	Delivery Efficiency ^h^ (%)	Topical Selectivity ^i^ (%)
Untreated	0.00 ± 0.00	NA ^j^	0.00 ± 0.00	NA^j^	0.00 ± 0.00	0.00 ± 0.00	2.53 ± 0.48	2.53 ± 0.48	100.00 ± 0.00
Base-treated	0.00 ± 0.00	NA ^j^	0.00 ± 0.00	NA^j^	0.00 ± 0.00	0.00 ± 0.00	2.60 ± 0.45	2.60 ± 0.45	100.00 ± 0.00
PLGA 50-2A	6.13 ± 1.61	4.16 ± 0.74	0.34 ± 0.05	3.88 ± 0.66	3.40 ± 0.49	311.58 ± 45.23	9.44 ± 1.59	9.44 ± 1.59	35.86 ± 7.37
PLGA 50-8A	39.25 ± 10.89	1.33 ± 0.42	1.69 ± 0.50	11.85 ± 4.55	16.90 ± 5.04	988.92 ± 294.85	44.15 ± 10.62	44.15 ± 10.62	11.60 ± 3.36

^a^ Cumulative amount (Q_24_) of MTX permeated through the unit diffusion area in 24 h. ^b^ Lag time (T_lag_)—calculated as the x-intercept of the linear portion of the permeation curve (R^2^ > 0.95). ^c^ Steady-state flux (Jss)—calculated from the linear slope of the permeation curve. ^d^ Diffusion coefficient (D)—calculated using an equation: D = h^2^/6t, where D is diffusion coefficient (sq·cm/h), h is skin thickness (cm), and t is lag time (h). ^e^ Permeability coefficient (K_p_)—calculated using an equation: K_p_ = J/CA, where K_p_ is the permeability coefficient (cm/h), J is the steady-state flux (µg/h), C is the MTX concentration in the donor (µg/mL), and A is the permeation area [8]. ^f^ Steady-state plasma concentration (C_ss_)—calculated using an equation: C_ss_ = (A × J_ss_)/Cl, where C_ss_ is the steady-state plasma concentration (µg/mL), A is the permeation area of skin (0.64 sq·cm), J_ss_ is the steady-state flux (µg/sq·cm/h), and Cl is the clearance of vismodegib from the body. ^g^ Total delivery is the total drug amount delivered into and across skin. ^h^ Delivery efficiency is the percentage of drug delivery to the amount of drug applied on skin. ^i^ Topical selectivity is the percentage of the drug level deposited in skin layers to the total drug delivery. ^j^ Not applicable.

## Data Availability

Data are contained within the article.

## Abbreviations

FTIR	Fourier-Transform Infrared Spectroscopy
MTX	Methotrexate
PBS	Phosphate-Buffered Saline
PPI	Pore Permeability Index
RP-HPLC	Reversed-Phase High-Performance Liquid Chromatography
SD	Standard Deviation
SEM	Scanning Electron Microscopy
TEWL	Transepidermal Water Loss
